# An asymmetric synthesis of all stereoisomers of piclavines A1-4 using an iterative asymmetric dihydroxylation

**DOI:** 10.1186/1860-5397-3-37

**Published:** 2007-10-29

**Authors:** Yukako Saito, Naoki Okamoto, Hiroki Takahata

**Affiliations:** 1 Faculty of Pharmaceutical Sciences, Tohoku Pharmaceutical University, Sendai 981-8558, Japan

## Abstract

The asymmetric synthesis of both enantiomers of piclavines A1, A2, A3, and A4 has been achieved using an iterative asymmetric dihydroxylation with enantiomeric enhancement.

## Background

**Figure 1:**
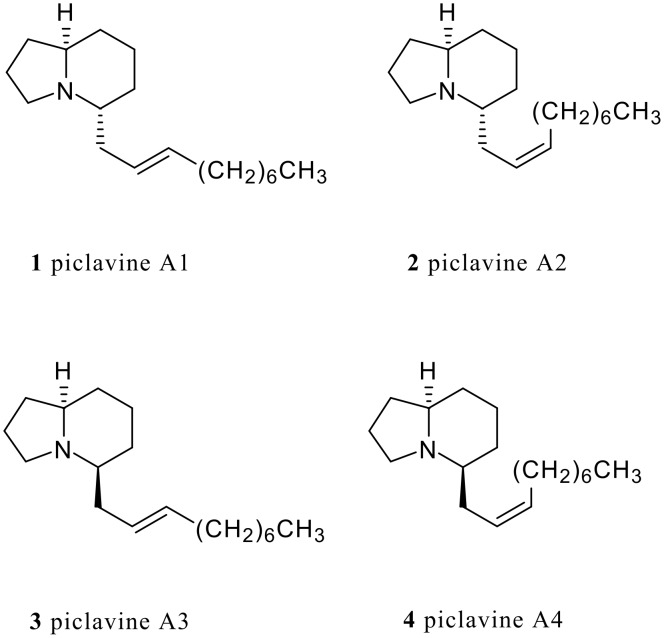
Piclavines A1-A4.

Indolizidine units are frequently found in many natural products and designed bioactive molecules. [1] Among these alkaloids, piclavines A1-4 (Figure 1), extracted from the tunicate *Clavelina picta* and the first indolizidine alkaloids to be found in the marine biosphere, exhibit interesting antimicrobial activities. [2] However, very little effort has been made to synthesize the piclavines. So far, among the four isomers shown in Figure 1, the synthesis of piclavine A4 [3] and a mixture of piclavines A1 and A2 [4] has been reported, but the synthesis of all four isomers has never been reported. In addition, their biological activities have been evaluated as a mixture of piclavines A1-4. [2] Therefore, we were inspired to develop a comprehensive synthetic program for these alkaloids.

**Scheme 1:**
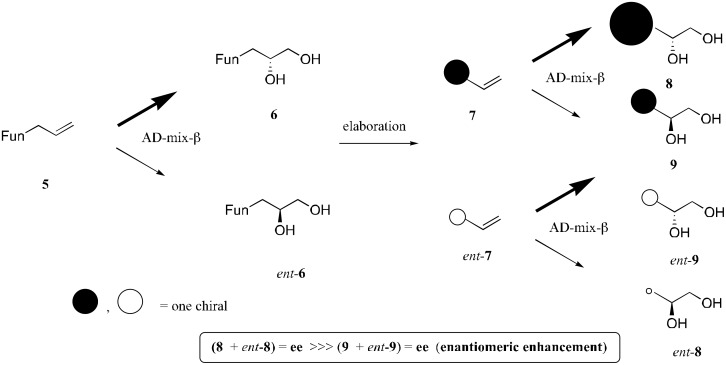
Enantiomeric enhancement by iterative AD.

The asymmetric synthesis of an indolizidine ring remains a great challenge. Our interest in this field has been focused on potential strategies based on the enantiomeric enhancement caused by the twofold or more application of the Sharpless asymmetric dihydroxylation (AD) [5–6] or Brown's asymmetric allylboration[7] reactions. In general, the enantiomeric excesses (ees) obtained for AD of terminal olefins are lower than for *trans* disubstituted olefins. However, it is expected that iterative AD terminal olefins will give products with high ees based on the following consideration. The first AD (AD-mix-β) of **5** produces major and minor enantiomers, **6** and *ent*-**6**, which are elaborated by introduction of terminal olefins to afford **7** and *ent*-**7**, respectively. The second AD of a mixture of **7** and *ent*-**7** provides four products **8**, **9**, *ent*-**9**, and *ent*-**8**. The relationship between **8** and **9** is diastereomeric. Since very little of the mirror image compound *ent*-**8** is prepared, the ee of the major product **8** will be very high. On the other hand, the ee of the minor diastereomer **9** or *ent*-**9** will be a low (Scheme 1). In most cases, when the products prepared by the iterative AD are acyclic and their asymmetric centers are remote, it is difficult to separate two diastereomers. Since transformation of acyclic compounds to cyclic derivatives provides rigid conformation and causes close proximity between two chiral centers, it is expected to greatly facilitate separation of two diastereomers. In this line, we report a full paper describing a new synthesis of all stereoisomers of piclavines A1-4 with high enantiomeric purity (amplification) for the major diastereomer via iterative AD reaction of terminal olefins. [8]

## Results

**Scheme 2:**
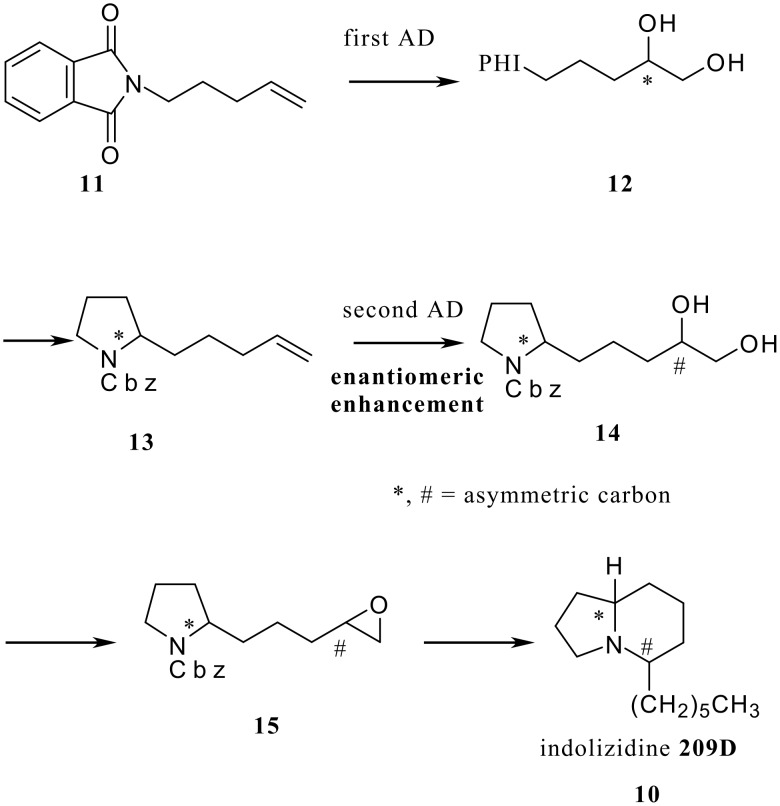
Asymmetric synthesis of indolidines **209D** using an iterative AD.

Actually we developed a general access to 5-substituted indolizidines **10** (all four stereoisomers of indolizidine 209D) with high enantio-enhancement (92–98% ee) via a sequence of iterative AD reactions starting from an achiral *N-*pentenylphthalimide (**11**). [9] The two stereogenic centers in **10** were constructed with high enantio-enhancement via a sequence of twofold AD reactions as shown in Scheme 2.

**Scheme 3:**
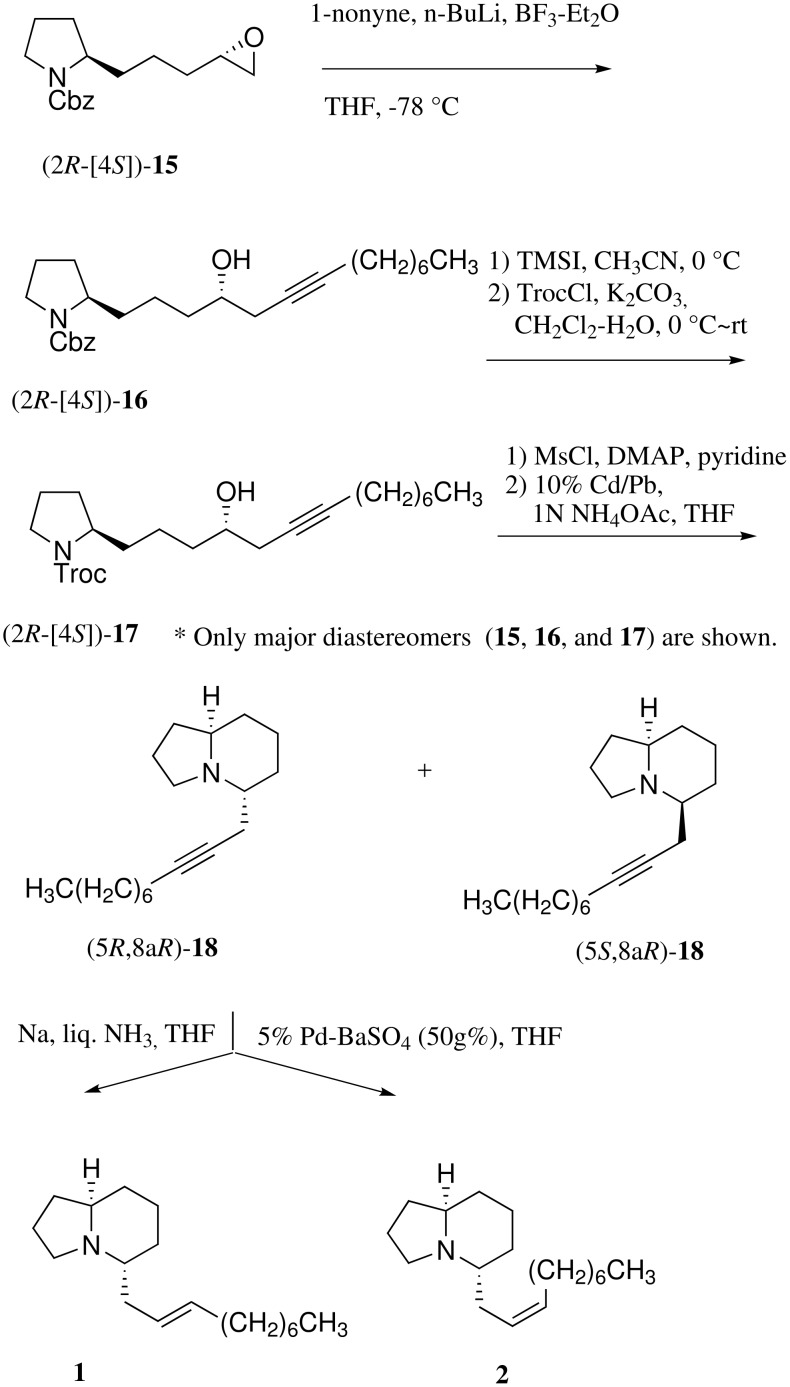
Asymmetric synthesis of piclavines A1 and A2 from (2*R*-[4*S*])-**15**.

We embarked on the synthesis of piclavines A1-4 using the four stereoisomers of the epoxides **15**[9] derived from **11** according to our reported procedure as synthetic intermediates. Regioselective cleavage of the epoxide (2*R*-[4*S*])-**15** rings with lithium acetylide generated from 1-nonyne with *n*-butyl lithium in combination with BF_3_-Et_2_O[10] gave the secondary alcohols (2*R*-[4*S*])-**16** in 94%. It is impossible to utilize hydrogenolysis due to reactivity of the acetylene unit. Indeed, the *N*-protecting group exchange of benzyloxycarbonyl (Cbz) for 2,2,2-trichloroethoxycarbonyl (Troc) in **17** was examined. The use of basic reagents such as Ba(OH)_2_[11] and KOH[12] failed to afford clean deprotection of Cbz. However, treatment of (2*R*-[4*S*])-**16** with iodotrimethylsilane (TMSI)[13] in CH_3_CN provided the amine (75%), which was treated with TrocCl/K_2_CO_3_ to afford the Troc carbamates (2*R*-[4*S*])-**17**. After mesylation of [2*S*-(4*R*)]-**17**, *N*-deprotection of the resulting mesylate with 10% Cd-Pb[14] gave the desired (5*R*,8a*R*)-**18** {[α]^25^_D_ +9.03 (*c* 0.32, CH_3_OH)} as a major product and (5*R*,8a*S*)-**18** {[α]^25^_D_ −22.7 (*c* 1.54, CH_2_Cl_2_)} as a minor product in a ratio of 2.3:1 in 25% overall yield. As expected, at this stage it was possible to separate the two diastereomers because transformation of monocyclic compounds (pyrrolidines) to bicyclic derivatives (indolizidines) provides rigid conformation and causes close proximity (a change from 1,5- to 1,3-relationship) between the two asymmetric centers. With indolizidine (5*R*, 8a*R*)-**18** in hand, we examined partial-reduction of their triple bonds. First, treatment of (5*R*, 8a*R*)-**18** with sodium in liquid ammonia gave the desired piclavine A1 (**1**) {[α]^25^_D_ −5.6 (*c* 0.84, CH_2_Cl_2_)} containing a *trans*-olefin in 71% yield. Exposure of hydrogen to (5*R*, 8a*R*)-**18** in the presence of Lindlar catalyst (Pd/CaCO_3_/Pb) or Rosenmund catalyst (5% Pd-BaSO_4_) was carried out in order to obtain a *cis* olefin product. However, hydrogenation using 10 g% catalysts scarcely proceeded, because the tertiary amine in indolizidine presumably works as a poison of the catalyst. Accordingly, the use of a large amount (50 g%) of 5% Pd-BaSO_4_ took place with semi-reduction of (5*R*,8a*R*)-**18** to provide piclavine A2 (**2**) {[α]^25^_D_ +4.03 (*c* 0.21 CH_2_Cl_2_)} in 53% yield (Scheme 3). The ^1^H and ^13^C NMR spectra of synthetic piclavines A1 and A2 are in good agreement with those of a mixture of natural piclavines A1 and A2. [2]

**Scheme 4:**
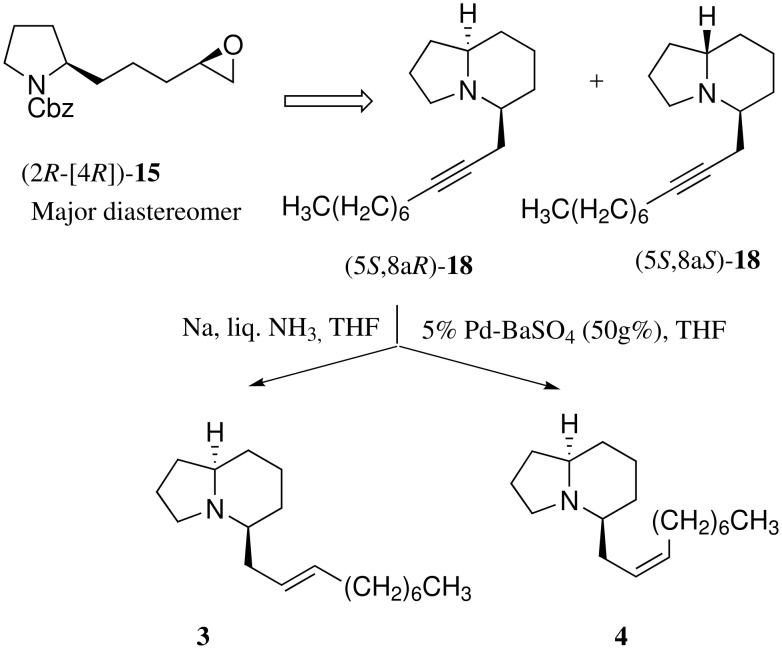
Asymmetric synthesis of piclavines A3 and A4 from (2*R*-[4*R*])-**15**.

A similar sequence of the epoxide (2*R*-[4*R*])-**15** prepared by (DHQD)_2_-PYR ligand-induced AD reaction of (*R*)-**13** afforded the desired (5*S*,8a*R*)-**18** {[α]^25^_D_ −67.5 (*c* 1.11, CH_2_Cl_2_)} as a major product and (5*S*,8a*S*)-**18** {[α]^25^_D_ −3.11 (*c* 0.62, CH_3_OH)} as a minor product in a ratio of 3.6:1 in 46% overall yield from (2*R*-[4*R*])-**15**. Similar semi-reduction of (5*S*, 8a*R*)-**18** with Na/NH_3_ and H_2_/10%Pd-BaSO_4_ gave piclavine A3 (**3**) (76%) {[α]^25^_D_ −74.3 (*c* 1.30, CH_2_Cl_2_) and piclavine A4 (**4**) [α]^25^_D_ −76.5 (*c* 0.63 CH_2_Cl_2_)} lit. [3] [α]^20^_D_ −74.8 (*c* 0.5 CH_2_Cl_2_)} in 84% yield, respectively (Scheme 4). Spectral data of **4** were completely consistent with values reported. [3]

**Scheme 5:**
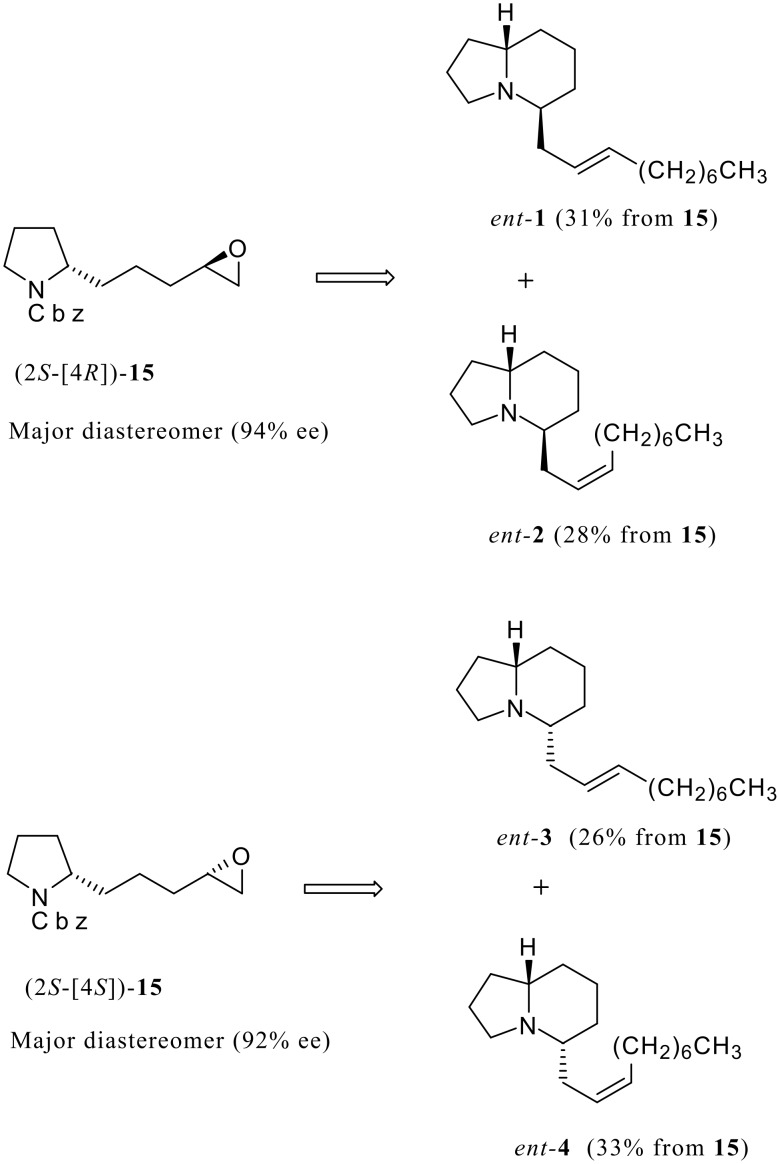
Asymmetric synthesis of enantiomers of piclavines (**1–4**).

With two diastereomers (2*S*-[4*R*])- and (2*S*-[4*S*])-**15** in hand, [9] the enantiomers of piclavines A1-4 were prepared according to the procedure described above (Scheme 5). However, the absolute configuration of natural products remains unassigned. [2]

## Conclusion

In summary, we accomplished the asymmetric synthesis of both enantiomers of piclavines A1, A2, A3, and A4 with high enantio-enhancement via iterative AD reactions starting from an achiral *N-*pentenylphthalimide **11**.

## Supporting Information

File 1 Synthetic details, spectral properties and HRMS data. Experimental details for an asymmetric synthesis of all stereoisomers of piclavines A1-4 using an iterative asymmetric dihydroxylation. Experimental data which includes experimental details on the spectral instruments.
