# Supplementary material for: An asymmetric synthesis of all stereoisomers of piclavines A1-4 using an iterative asymmetric dihydroxylation
Source: Beilstein J Org Chem. 2007 Oct 29;3:37. doi: 10.1186/1860-5397-3-37 (PMC2200665; doi:10.1186/1860-5397-3-37)
Supplement: File 1 — Synthetic details, spectral properties and HRMS data. Experimental details for an asymmetric synthesis of all stereoisomers of piclavines A1-4 using an iterative asymmetric dihydroxylation. Experimental data which includes experimental details on the spectral instruments. [file Beilstein_J_Org_Chem-03-37-s001.doc]

# General

Melting points were determined with a Yanaco micro melting point apparatus and are uncorrected. ^1^H and ^13^C NMR spectra were taken on a Varian Gemini 300 or JEOL JNM-AL 400 spectrometer. ^1^H NMR spectra were recorded at the indicated field strength as solutions in CDCl_3_ unless otherwise indicated. Chemical shifts are given in parts per million (ppm, δ) downfield from TMS and are referenced to CHCl_3_ (7.26 ppm) as an internal standard. Splitting patterns are designated as s, singlet; d, doublet; t, triplet; q, quartet; m, multiplet; br, broad. ^13^C NMR spectra were recorded at the indicated field strength as solutions in CDCl_3_ unless otherwise indicated. Chemical shifts are given in ppm, (δ) downfield from TMS and are referenced to the center line of CDCl_3_ (77.0 ppm) as an internal standard. Infrared spectra (IR) were measured with a Perkin-Elmer 1600 series FT-IR spectrophotometer. Mass spectra (MS) and high-resolution mass spectra (HRMS) were measured on a JEOL JMS-AX505HAD or a JEOL JMN-DX 303/JMA-DA 5000 mass spectrometer. Optical rotations were measured on a JASCO DIP-1000 or JASCO DIP-360 or JASCO P-1020 digital polarimeter. Column chromatography was performed with Kanto Kagaku silica gel 60N.

**(*R*)-Benzyl 2-((*S*)-4-hydroxytetradec-6-ynyl)pyrrolidine-1-carboxylate (2*R*-[4*S*])-16 and its diastereomer.**

To a stirred solution of 1-nonyne (0.5 mL, 2.84 mmol) in THF (1.1 mL) was added 1.6 M *n*-BuLi (1.87 mL, 2.84 mmol) in *n*-hexane at -78 °C. After being stirred for 10 min, BF_3_–Et_2_O (0.45 mL, 2.84 mmol) was added to the resulting solution at -78 °C. After being stirred for 10 min, (2*R*-[4*S*])-**15** (97% ee) [9] (548 mg, 1.89 mmol) in THF (3.0 mL) was successively added to the mixture at -78 °C, and the resulting solution was stirred at the same temperature for 30 min. After addition of saturated NH_4_Cl wit ice cooling, the mixture was extracted with ethyl acetate three times. The extracts were washed with brine, dried over anhydrous Na_2_SO_4_, and evaporated to give a colorless oil, which was chromatographed on silica gel (*n*-hexane : ethyl acetate = 4 : 1) to afford a diastereomeric mixture of **16** ((2*R*-[4*S*])-**16** as a major diastereomer) (737mg, 94%) as an oil.

IR (neat) 3446, 2930, 1684, 1653 cm^-1^; ^1^H NMR (300 MHz) δ 0.88 (3H, d, *J* = 6.9 Hz), 1.23-2.34 (25H, m), 3.33-3.41 (2H, m), 3.60-3.68 (1H, m), 3.85 (1H, br s), 5.06-5.12 (2H, m) 7.27-7.37 (5H, m); ^13^C NMR (75 MHz) δ 14.28, 18.94, 22.19, 22.57, 22.72, 22.81, 23.21, 23.95, 27.99, 28, 08, 28.97, 29.03, 29.17, 30.06, 3073, 31.90, 33.98, 34.72, 36.04, 36.15, 46.36, 57.37, 57.87, 66.58, 66.74, 69.98, 70.05, 70.23, 76.14, 76.32, 83.07, 127.80, 128.39, 137.02, 154.93. HRMS calcd for C_26_H_39_NO_3_ 413.2930, found 413.2945.

**(*R*)-Benzyl 2-((*R*)-4-hydroxytetradec-6-ynyl)pyrrolidine-1-carboxylate (2*R*-[4*R*])-16 and its diastereomer.**

According to the analogous procedure described for (2*R*-[4*S*])-**16** and its diastereomer, a mixture of (2*R*-[4*R*])-**15** (98% ee) [9] (1.90 g, 6.57 mmol), 1-nonyne (1.74 mL, 9.86 mmol), 1.6 M *n*-BuLi (6.16 mL, 9.86 mmol) in *n*-hexane, and BF_3_–Et_2_O (1.57 mL, 9.86 mmol) afforded a diastereomeric mixture of **16** ((2*R*-[4*R*])-**16** as a major diastereoer) (2.06 g, 96%) as an oil. Spectral data are almost the same as those of the above **16** ((2*R*-[4*S*])-**16** as a major diastereomer).

**(*R*)-2,2,2-Trichloroethyl 2-((*S*)-4-hydroxytetradec-6-ynyl)pyrrolidine-1-carboxylate (2*R*-[4*S*])-17 and its diastereomer.**

To a solution of a diastereomeric mixture of **16** ((2*R*-[4*S*])-**16** as a major diastereomer) (737 mg, 1.78 mmol) in CH_3_CN (37.4 mL) was slowly added iodo trimethylsilane (TMSI) (0.52 mL, 3.56 mmol) with ice cooling, and the resulting mixture was stirred for 30 min at 0 °C. After evaporation, 10% HCl was added to the residue with ice cooling. The mixture was washed with ether. To the aqueous layer was added 10%KOH, and the resulting basic mixture was extracted with CH_2_Cl_2_. The extract was washed with H_2_O, and dried over anhydrous MgSO_4_ and evaporated to give a colorless oil, which was chromatographed on alumina (ethyl acetate: CH_3_OH = 10 : 1) to afford a diastereomeric mixture of secondary amine (385mg, 77%) as a pale yellow oil. To a stirred solution of the amine (385 mg, 1.38 mmol) obtained above in CH_2_Cl_2_ (10.9 mL) and H_2_O (3.6 mL) were successively added K_2_CO_3_ (287 mg, 2.07 mmol) and chloroformic acid 2,2,2-trichloroethyl ester (TrocCl) (0.21 mL, 1.52 mol) at 0 °C, and the resulting mixture was stirred at room temperature for 1h. The mixture was separated, and the aqueous layer was extracted with CH_2_Cl_2_. The organic layers were combined, dried over anhydrous Na_2_SO_4_, and evaporated to give a colorless oil, which was chromatographed on silica gel (*n*-hexane : ethyl acetate = 5 : 1) to afford a diastereomeric mixture of **17** ((2*R*-[4*S*])-**17** as a major diastereomer) (417 mg, 67%) as a pale yellow oil,

IR (neat) 3446, 2929, 2363, 1718, 1647 cm^-1^; ^1^H NMR (300 MHz) δ 0.88 (3H, d, *J* = 6.9 Hz), 1.28-1.53 (16H, m), 1.70-2.02 (6H, m), 2.13-2.42 (4H, m), 3.39-3.53 (2H, m), 3.64-3.70 (1H, m), 3.90 (1H, br s), 4.60-4.90 (2H, m); ^13^C NMR (75 MHz) δ 14.32, 18.97, 22.22, 22.49, 22.64, 22.72, 22.84, 23.28, 23.92, 28.02, 28.38, 29.02, 29.06, 39/20, 300.9, 30.64, 31.95, 33.60, 33.75, 34.42, 34.62, 36.10, 36.27, 46.56, 47.05, 57.81, 57.91, 58.2, 70.04, 70.16, 70.23, 74.77, 74.97, 76.02, 76.15, 83.36, 83.50, 95.87, 96.06, 153.02; MS: 453(M^+^); HRMS calcd for C_21_H_34_Cl_3_NO_3_ 453.1604, found 453.1571.

**(*R*)-2,2,2-Trichloroethyl 2-((*R*)-4-hydroxytetradec-6-ynyl)pyrrolidine-1-carboxylate (2*R*-[4*R*])-17 and its diastereomer.**

According to the analogous procedure described for (2*R*-[4*S*])-**17** and its diastereomer, a mixture of (2*R*-[4*R*])-**16** (1.35 g, 4.83 mmol), TMSI (1.83 mL, 12.6 mmol) in CH_3_CN (132 mL) afforded a diastereomeric mixture of the amine (1.35 g, 77%) as a pale yellow oil, which was used directly in the next step. A mixture of the amine (1,35 g, 4.83 mmol) and K_2_CO_3_ (1.01 g, 7.25 mmol), and TrocCl (0.73 mL, 5.32 mmol) in CH_2_Cl_2_ (38 mL) and H_2_O (12.7 mL) provided a diastereomeric mixture of **17** ((2*R*-[4*R*])-**17** as a major diastereomer) (1.80 g, 82%) as an oil. Spectral data are almost the same as those of the above **17** ((2*R*-[4*S*])-**17** as a major diastereomer).

**(5*R*,8a*R*)-5-(Dec-2-ynyl)-octahydroindolizidine (5*R*,8a*R*)-18 and (5*S*,8a*R*)-5-(Dec-2-ynyl)-octahydroindolizidine (5*S*,8a*R*)-18.**

To a stirred solution of **17** ((2*R*-[4*S*])-**17** as a major diastereomer) (411 mg, 0.904 mmol) and DMAP (25 mg, 0.130 mmol) in pyridine (18.1 mL) was added methanesulfonyl chloride (MsCl) (0.10 mL, 1.35 mmol) with ice cooling, and the resulting mixture was stirred for 2.5 h at room temperature. After addition of ether to the reaction mixture, the resulting mixture was acidified with 20% KHSO_4_ with ice cooling. The mixture was successively washed with H_2_O and brine, dried over anhydrous Na_2_SO_4_, and evaporated to afford the mesylate, which was used directly in the next step. To the residue in THF (7.2 mL) were successively added 1N NH_4_OAc (7.2 mL) and 10%Cd/Pb (1.20 g, 9.04 mmol), and the resulting mixture was stirred overnight at room temperature. After being diluted with CH_2_Cl_2_, the resulting mixture was basified with 2N NaOH and stirred for 5 min. The mixture was filtered on Celite, and the filtrate was separated. The aqueous layer was washed with CH_2_Cl_2_, and the combined organic solvents were dried over anhydrous K_2_CO_3_ and evaporated. The residue was chromatographed on silica gel (*n*-hexane : ethyl acetate = 10 : 1) to afford (5*S*,8a*R*)-**18** (53 mg, 23%) and (5*R*,8a*R*)-**18** (122 mg, 52%) as oils.

(5*S*,8a*R*)-**18:** [α]^26^_D_ = -22.7 (*c* 1.54, CH_2_Cl_2_); IR (neat) 2929, 2856, 2783, 1437 cm^-1^; ^1^H NMR (300 MHz) δ 0.90 (3H, t, *J* = 7.05 Hz), 1.19-1.53(11H, m), 1.65-1.93 (8H, m), 2.03-2.18 (6H, m), 2.58-2.62 (1H, m), 3.19 (1H, td, *J* = 2.20, 8.52 Hz); ^13^C NMR (75 MHz) δ 14.37, 19.06, 20.72, 22.87, 24.74, 25.41, 29.06, 29.31, 30.67, 31.17, 31.72, 32.01, 51.83, 63.42, 65.12, 77.87, 82.03; HRMS calcd for C_18_H_31_N (M^+^) 261.2456, found 261.2435.

(5*R*,8a*R*)-**18**: [α]^27^_D_ = 9.03 (*c* 0.32, CH_3_OH), [α]^26^_D_ = 1.03 (*c* 0.61, CH_2_Cl_2_); IR (neat) 2928, 2856, 2799, 1458 cm^-1^; ^1^H NMR (300 MHz) δ 0.88 (3H, t, *J* = 6.59 Hz), 1.08-1.96 (19H, m), 2.10-2.46 (6H, m), 2.62 (1H, q, *J* = 8.79 Hz), 2.89 (1H, td, *J* = 2.75, 8.79 Hz), 3.20-3.27 (1H, m); ^13^C NMR (75 MHz) δ 14.02, 14.40, 19.12, 19.44, 21.10, 22.90, 27.94, 29.09, 29.37, 30.90, 31.61, 32.04, 49.28, 55.27, 55.62, 78.89, 81.77; HRMS calcd for C_18_H_31_N (M+) 261.2456, found 261.2458.

**(5*S*,8a*R*)-5-(Dec-2-ynyl)-octahydroindolizidine (5*S*,8a*R*)-18 and (5*R*,8a*R*)-5-(Dec-2-ynyl)-octahydroindolizidine (5*S*,8a*S*)-18.**

According to the analogous procedure described for (5*R*,8a*R*)-**18** and (5*S*,8a*R*)-**18**, a mixture of **17** ((2*R*-[4*R*])-**17** as a major diastereomer) (76 mg, 0.167 mmol), DMAP (4.5 mg, 0.025 mmol), and MsCl (0.019 mL, 0.251 mmol) in pyridine (3.34 mL) afforded the mesylate, which was cyclized with 1N NH_4_OAc (1.33 mL) and 10% Cd/Pb (222 mg, 1.67 mmol) in THF (1.33 mL) to provide (5*S*,8a*R*)-**18** (21 mg, 46%) and (5*S*,8a*S*)-**18** (5.8 mg, 13%) as oils. Spectral data of (5*S*,8a*R*)-**18** and (5*S*,8a*S*)-**18** were identical with those of (5*S*,8a*R*)-**18** and (5*R*,8a*R*)-**18** described above, respectively.

(5*S*,8a*R*)-**18**: [α]^25^_D_ = –67.5 (*c* 1.11, CH_2_Cl_2_); (5*S*,8a*S*)-**18**: [α]^27^_D_ = –3.11 (*c* 0.62, CH_3_OH).

**(5*R*,8a*R*)-5-((*E*)-Dec-2-enyl)-octahydroindolizine (piclavine A1) (1).**

To liquid NH_3_ (20 mL) was added Na (175 mg, 7.6 mmol) at –78 °C, the resulting mixture was stirred for 30 min. A solution of (5*R*,8a*R*)-**18** (31 mg, 0.119 mmol) in THF was added to the mixture at –60 °C and the whole was refluxed for 3.5 h. After addition of saturated NH_4_OAc, the resulting mixture was diluted with CH_2_Cl_2_. The mixture was separated and the aqueous layer was extracted with CH_2_Cl_2_, The combined organic solvents were washed with brine, dried over anhydrous K_2_CO_3_, and evaporated. The residue was chromatographed on silica gel (*n*-hexane : ethyl acetate = 10 : 1) to afford **1** (22 mg, 71%) as a colorless oil; [α]^26^_D_ -5.60 (*c* 0.84, CH_2_Cl_2_); IR (neat) 2925, 2853, 1458, 967, 754 cm^−1^; ^1^H NMR (300 MHz) δ 0.87 (3 H, t, *J*=6.59 Hz), 1.08–1.85 (17H, m), 1.94–2.11 (3H, m), 2.23–2.29 (1H, m), 2.38–2.48 (1H, m), 2.65 (1H, q, *J*=8.79 Hz), 2.83 (1H, td, *J*=3.11, 8.65 Hz), 2.96–3.02 (1H, m), 5.25–5.46 (2H, m); ^13^C NMR (75 MHz) δ 14.43, 19.44, 21.14, 22.95, 26.73,27.32, 29.40, 29.44, 29.84, 31.01, 31.70, 32.13, 32.96, 49.60, 55.30, 55.74, 128.33, 132,35; HMRS calcd for C_18_H_33_N (M^+^) 263.2613, found 263.2585.

**(5*R*,8a*R*)-5-((*Z*)-Dec-2-enyl)-octahydroindolizine (piclavine A2) (2).**

A suspension of (5*R*,8a*R*)-**18** (37 mg, 0.142 mmol) and 5% Pd-BaSO_4_ (19 mg) in CH_3_OH (0.25 mL) under hydrogen was stirred at room temperature for 29 h. The reaction mixture was filtered on Celite, and the filtrate was evaporated. The residue was chromatographed on silica gel (*n*-hexane : ethyl acetate = 10 : 1) to afford **2** (19 mg, 52%) as a colorless oil; [α]^27^_D_ +4.03 (*c* 0.21, CH_2_Cl_2_); IR (neat) 2926, 2855, 803 cm^−1^; ^1^H NMR (300 MHz) δ 0.88 (3H, t, *J*=6.59 Hz), 1.12–1.86 (20H, m), 2.00–2.29 (4H, m), 2.45–2.47 (1H, m), 2.69 (1H, q, *J*=8.79 Hz), 2.86 (1H, td, *J*=3.30, 8.79 Hz), 2.99–3.04 (1H, m), 5.25–5.46 (2H, m); ^13^C NMR (75 MHz) δ 14.42, 1 9.55, 21.10, 22.96, 27.41, 27.71, 29.09, 29.50, 29.59, 29.97, 30.76, 32.13, 49.16, 55.65, 56.02, 127.477, 131.514; HRMS calcd for C_18_H_33_N (M^+^) 263.2613, found 263.2624.

**(5*S*,8a*R*)-5-((*E*)-Dec-2-enyl)-octahydroindolizine (piclavine A3) (3).**

According to the analogous procedure described for **1**, a mixture of (5*S*,8a*R*)-**18** (100 mg, 0.382 mmol), Na (313 mg, 13.6 mmol) in liquid NH_3_ (30 mL) afforded **3** (77 mg, 76%) as a colorless oil; [α]^26^_D_ -73.6 (*c* 1.30, CH_2_Cl_2_); IR (neat) 2926, 2854, 2780, 1458, 1437, 1129, 968 cm^−1^; ^1^H NMR (300 MHz) δ 0.87 (3H, t, *J*=6.59 Hz), 1.04–1.49 (13H, m), 1.56–2.02 (12H, m), 2.38–2.44 (1H, m), 3.26 (1H, td, *J*=2.20, 8.52 Hz), 5.36–5.47 (2H, m); ^13^C NMR (75 MHz) δ 14.42, 20.73, 22.95, 24.89, 29.41, 29.44, 29.84, 30.76, 31.25, 32.13, 32.93, 38.56, 51.90, 64.10, 65.26, 127.11, 132.74; HRMS calcd for C_18_H_33_N (M^+^) 263.2613, found 263.2634.

**(5*S*,8a*R*)-5-((*Z*)-Dec-2-enyl)-octahydroindolizine (piclavine A4) (4).**

A suspension of (5*S*,8a*R*)-**18** (44 mg, 0.168 mmol) and 5% Pd-BaSO_4_ (22 mg) in CH_3_OH (0.26 mL) under hydrogen was stirred at room temperature for 24 h. The reaction mixture was filtered on Celite, and the filtrate was evaporated. The residue was chromatographed on silica gel (*n*-hexane : ethyl acetate = 10 : 1) to afford **4** (37 mg, 84%) as a colorless oil; [α]^27^_D_ -76.4 (*c* 0.63, CH_2_Cl_2_); IR (neat) 2926, 2854, 2780, 754 cm^−1^; ^1^H NMR (300 MHz) δ 0.87 (3 H, t, *J*=6.32 Hz), 1.07–1.50 (14H, m), 1.61–2.12 (12H, m), 2.36–2.44 (1H, m), 3.25–3.31 (1H, m), 5.31–5.47 (2H, m); ^13^C NMR (75 MHz) δ 14.42, 20.75, 22.95, 24.92, 27.72, 29.47, 29.59, 29.91, 30.81, 31.28, 31.34, 32.14, 33.02, 51.92, 64.09, 65.24, 126.52, 131.59; HRMS calcd for C_18_H_33_N (M^+^) 263.2613, found 263.2591.

**Synthesis of enantiomers of 1-4.**

According to the analogous procedure described for **1-4**, *ent*-**1** [α]^26^_D_ 5.48 (*c* 0.59, CH_2_Cl_2_); and *ent*-**2**  [α]^26^_D_ –3.98 (*c* 0.63, CH_2_Cl_2_);, were obtained from (2*S*-[4*R*])-**15** (94% ee) [9] and *ent*-**3** [α]^26^_D_ 72.5 (*c* 0.86, CH_2_Cl_2_); and *ent*-**4** [α]^26^_D_ 74.4 (*c* 0.43, CH_2_Cl_2_) were prepared from (2*S*-[4*S*])-**15** (92% ee) [9].
